# Flexible SERS substrate of silver nanoparticles on cotton swabs for rapid *in situ* detection of melamine

**DOI:** 10.1039/d1na00670c

**Published:** 2022-01-13

**Authors:** Wen-Chien Huang, Ken-Fa Cheng, Jing-Yuan Shyu

**Affiliations:** Department of Chemical and Materials Engineering, Chung Cheng Institute of Technology, National Defense University Taoyuan 33551 Taiwan wenchien2@gmail.com; Chemical Systems Research Division, National Chung-Shan Institute of Science and Technology Taoyuan 32599 Taiwan

## Abstract

It is important to be able to detect melamine *via* a sensitive and fast method in the field of food safety. Surface-enhanced Raman scattering (SERS) has attracted much attention due to its high sensitivity, rapid results, unique spectroscopic fingerprint, and nondestructive data acquisition. In this work, we describe the preparation of flexible CS-ATS-Ag cotton swabs for use in SERS by anchoring silver nanoparticles (AgNPs), as a highly-sensitive SERS material, on cotton swabs (CS) using *N*-[3-(trimethoxysilyl)propyl] diethylenetriamine (ATS) as the coupling agent. The flexible CS-ATS-Ag cotton swabs exhibited high SERS sensitivity, uniformity and reproducibility as a melamine molecule probe, and the limit of detection was calculated to be 0.2 ppm. A high SERS signal reproducibility was achieved, and the relative standard deviation (RSD) of the melamine peak at 699 cm^−1^ was approximately 5.01%. Moreover, we successfully developed Chemical analysis App application software; a smartphone was used to convert data and record the results, then the data were geotagged using the GPS feature in the smartphone and uploaded to a central website. The goal of realizing instant transmission, timely processing, high sensitivity, portability and low cost was therefore achieved.

## Abstract 2

Schematic illustration of the fabrication process of the SERS cotton swabs. Cell phones can be used for data collection and to push data to a website where data is displayed on a map.

## Graphical abstract

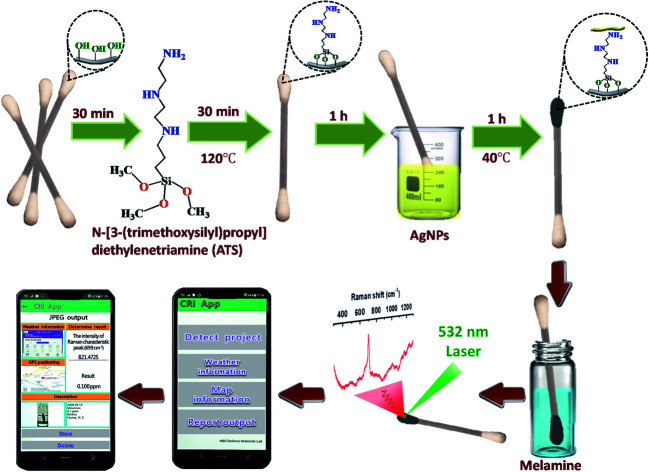

## 1. Introduction

Melamine is a chemical compound that is mainly used for resin production, thermosetting plastic and polymer manufacturing in general, and is widely used as a raw material in industry for producing synthetic polymers.^1,2^ As a rich-nitrogen molecule, melamine was intentionally added into food ingredients to produce an incorrectly high reading in the measurement of the protein content based on total nitrogen. Since 2007, owing to melamine's high nitrogen content (66% by mass), it has been illegally added to pet food and infant formula, which has attracted much attention. Kidney disease and even death in infants and pets due to ingestion of melamine have been recorded. Considering its potential toxicity, the Codex Alimentarius Commission has set limits for powdered infant formula (1 mg L^−1^) and other foods and animal feed (2.5 mg L^−1^).^3–5^

Recently, some conventional technologies, such as high-performance liquid chromatography (HPLC),^6,7^ gas chromatography-tandem mass spectrometry (GC-MS/MS)^8,9^ and enzyme-linked immunosorbent assay (ELISA),^10,11^ have been employed for the detection of melamine; however, these methods usually require expensive instruments, and lengthy sample preparation procedures are needed, mainly due to the analyte extraction steps. Surface-enhanced Raman scattering (SERS) has attracted much attention due to its high sensitivity, rapid results, unique spectroscopic fingerprint, and non-destructive analysis for molecule sensing, and has emerged as an up-and-coming technique for chemical and biosensing applications. SERS techniques have unique advantages in various fields, such as biomedicine,^12,13^ homeland security,^14,15^ environmental monitoring,^16–19^ and food safety.^20,21^ SERS is considered to be one of the most promising and powerful analytical techniques.

However, conventional SERS substrates were fabricated on hard supporting substrates such as glass, quartz and silicon. It is difficult to extract the target analytes from the irregular-shaped matrices before SERS detection owing to lack of flexibility of the SERS substrate. In conclusion, a low sample collection efficiency limits application for the detection of analytes on irregular matrices. Therefore, the development of flexible SERS substrates with high SERS activity, uniformity, reproducibility and stability became of increasing interest. For example, metal nanostructures have been deposited onto nanofibers,^22–24^ hydrogel,^25^ polystyrene-*block*-poly-(ethylene oxide),^26^ and polydimethylsiloxane^27,28^ to construct soft and flexible SERS substrates. In recent years, cotton swabs as a support material for the deposition of gold or silver nanoparticles have attracted considerable interest, owing to the low cost of the swabs and their ready accessibility and portability. SERS detection of analytes without the intricate pretreatment process required to obtain the analytes from irregular surfaces is one potential solution. Coupling agents on metal nanoparticles attach to various substrates through their terminal groups. The most commonly- and widely-used coupling agents include thiol-terminated, amine-terminated, alkyl-terminated and phenyl-terminated silane.

In this study, we present a simple method for the fabrication of excellent flexibility, highly-sensitive, portable and inexpensive SERS cotton swabs. The SERS cotton swabs with excellent flexibility and high robustness by anchoring AgNPs on cotton fibers using *N*1-(3-trimethoxysilylpropyl) diethylenetriamine (ATS) as the coupling agent. The silane coupling agent ATS forms covalent bonds on the CS surface *via* hydrolysis.^29^ At the interfaces between CS and silane groups, chemical bonding with –OH occurs on the CS surface. ATS can readily form a monomolecular layer on the CS surface. In the meantime, the presence of three amine groups in ATS can further bind metal ions.^30,31^ Strong water absorption and flexibility of cotton-based SERS cotton swabs enable it to rapidly capture target molecules from uneven samples by soaking and wiping. These flexible SERS cotton swabs were successfully used to detect melamine at a concentration as low as 0.2 ppm, and the reproducibility of the intensity of the SERS peaks was within 5.01%. More importantly, Chemical analysis App application software was successfully developed and used to convert data and record the results; the data were geotagged using the GPS feature in a smartphone and uploaded to a central website. The goal of realizing instant transmission, timely processing, high sensitivity, portability and low cost was therefore achieved.

## 2. Experimental

### 2.1. Materials

Sodium borohydride (NaBH_4_, Sigma-Aldrich Co., Ltd.) was used as a reducing agent. *N*-[3-(trimethoxysilyl)propyl]diethylenetriamine (ATS, Sigma-Aldrich Co., Ltd.) was used as a coupling agent. Melamine (Sigma-Aldrich Co., Ltd.) acted as the target detection molecule. Silver nitrate (AgNO_3_, Aldrich Co., Ltd.) was used to reduce AgNPs. Sodium citrate (TSC, Showa Co., Ltd.) was used as a protective agent. All chemical reagents used were of analytical grade and were used without further purification. Cotton swabs were purchased from Yuh Shiuans Co., Ltd. The water used in the experiment was ultrapure water (18.2 MΩ).

### 2.2. Apparatus

The UV-vis absorption spectrum of the silver nanoparticle (AgNP) solution was obtained using a ChromTech CT-8600 instrument (Chromtech-Analytical Instruments, Germany). The X-ray diffraction (XRD) patterns were recorded on a Bruker D2 Phaser diffractometer. Scanning electron microscope (SEM) images and the SEM-EDX spectrum of the SERS cotton swabs were recorded using a field-emission SEM (JSM-7600F, JEOL Ltd., Japan). SERS spectra were obtained using a Raman spectrometer (TRIAX 550, Horiba, Ltd., Japan) with an excitation wavelength of 532 nm. During detection, the integration duration of the Raman spectra was 10 s.

### 2.3. Preparation of AgNPs

AgNPs were synthesized by a simple chemical reduction method using sodium borohydride (NaBH_4_) as the reducing agent. An aqueous solution of AgNO_3_ (10 mL, 1 mM) was added to NaBH_4_ (30 mL, 4 mM) solution with stirring. The color of the mixed solution changed to brown, and the final color was bright yellow. Then, 1 mL of 0.02 M sodium citrate (TSC) was added to 40 mL of AgNP solution. TSC stabilized the AgNPs in a colloidal state owing to the repulsive force that existed among the particles, and this was maintained by a net negative charge on their surface.^32^ The above-mentioned procedure was then applied. All AgNP solutions were stored at 4 °C before use.

### 2.4. Fabrication of flexible CS-ATS-Ag cotton swabs

**Fig. 1:**
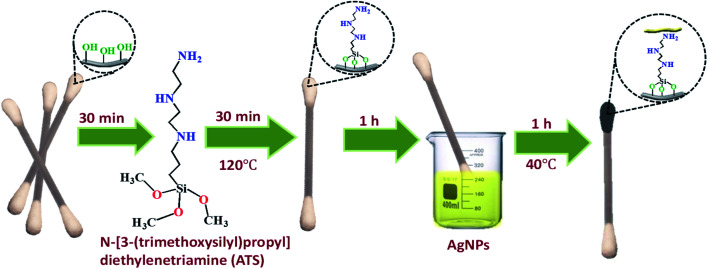
Schematic illustration of the fabrication process of the SERS cotton swabs.

Flexible CS-ATS-Ag cotton swabs were fabricated as follows. The cotton swabs were immersed in a 2% (v/v) ATS solution under ultrasonic agitation for 30 min. Then, they were removed from the ATS solution, rinsed with ultrapure water thoroughly, and dried in an oven at 120 °C for 30 min to activate the cotton fibers. The activated CS-ATS cotton swabs were soaked in AgNP solution under ultrasonic agitation for 1 h, following which the AgNP-modified cotton swabs were placed in a vacuum oven and heated at 40 °C for 1 h (Fig. 1).

### 2.5. Detection of melamine using the flexible CS-ATS-Ag cotton swabs

Melamine stock standard solution (1000 ppm) was prepared by dissolving melamine in ultrapure water, and was stored at 4 °C for further use. For sensitivity testing, melamine standard solutions were prepared by subsequent dilution from the stock solution in ultrapure water to attain the following concentrations: 5, 2.5, 1.25, 1, 0.7, 0.5 and 0.2 ppm. The flexible CS-ATS-Ag cotton swabs were immersed in 1 mL of melamine solution at various concentrations for 5 minutes. Finally, Raman spectra of the flexible CS-ATS-Ag cotton swabs were recorded using a Raman spectrometer.

For the reproducibility test, the flexible CS-ATS-Ag cotton swabs were immersed in 1 mL of melamine standard solution (5 ppm) for 5 minutes. Raman spectra of seven random spots on the flexible CS-ATS-Ag cotton swabs were recorded and averaged.

Two methods were used for stability testing. In the first method, the flexible CS-ATS-Ag cotton swabs were treated with ultrasonication for 30 min, and then taken out to observe whether the silver glue on the surface had fallen off. In the second method, the flexible CS-ATS-Ag cotton swabs were stored in a moisture-proof box for 2 months before testing, and the swabs were then immersed in 1 mL of melamine standard solution (5 ppm) for 5 minutes. The performance of the flexible CS-ATS-Ag cotton swabs after treatment was compared between methods.

## 3. Results and discussion

### 3.1. Characterization of the AgNPs

**Fig. 2:**
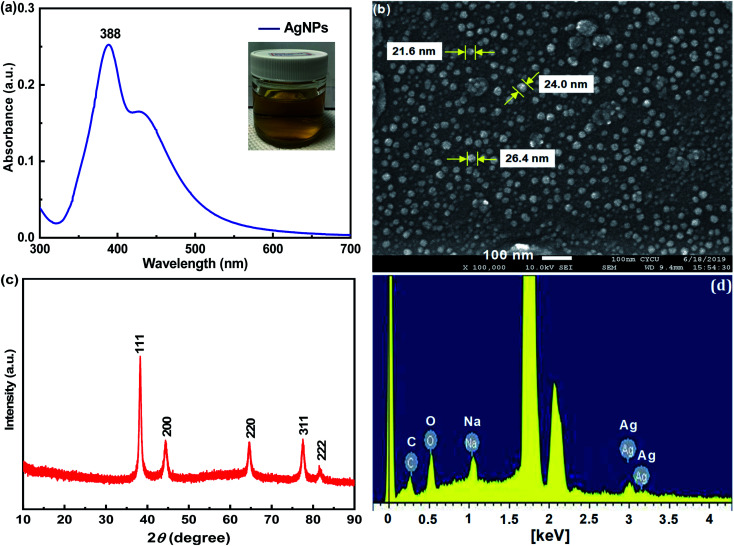
The colloidal AgNPs of (a) UV-vis spectrum, (b) SEM image, (c) XRD spectra and (d) EDS spectrum.

The dispersity and morphology of the AgNPs were characterized by UV-vis spectroscopy and SEM. A UV-vis spectrometer was employed to measure the absorbance of the colloidal AgNPs. It can be observed from Fig. 2(a) that the UV-vis spectrum of the AgNPs displayed a characteristic peak at about 388 nm. To further examine the morphology of AgNRs, SEM images were recorded. As shown in Fig. 2(b), the AgNRs were homogeneous in shape and size, and the TSC ligands on the surface of the AgNRs kept them well-separated. The AgNPs were of a spherical shape with a diameter distribution between 21 and 26 nm. Powder XRD was employed to verify the crystal structure and phase purity of the AgNPs, as shown in Fig. 2(c). Five peaks were observed at the 2*θ* positions 38.2°, 44.2°, 64.4°, 77.5° and 81.6°, corresponding to the (111), (200), (220), (311) and (222) planes of the face-centered cubic (fcc) AgNPs (JCPDS card no. 04-0783).^33^ In addition, no significant diffraction peaks arising from crystalline impurities were detected, indicating that the product was of a pure cubic Ag phase.^34^ The crystallite sizes were calculated using the Debye–Scherrer equation:1
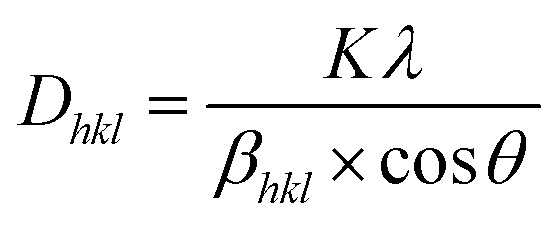
where *K* was the particle shape factor, *λ* was X-ray wavelength, *β*_*hkl*_ was the half-width of the (*hkl*) reflection, and *θ* was the Bragg angle corresponding to the (*hkl*) reflection.^35^ In this study, we employed the diffraction peak corresponding to the (111) crystal plane to calculate the average grain size (*D*) of the AgNPs, which was 21.01 nm.

The EDX profile showed strong signals for C, O and Ag, as shown in Fig. 2(d). Metallic AgNPs generally show a typical optical absorption peak at approximately 3 keV due to surface plasmon resonance.^36^ The signals of C and O may have originated from TSC bound to the surface of the AgNPs, indicating the presence of citrate protection.

### 3.2. Characterization of the flexible CS-ATS-Ag cotton swabs

**Fig. 3:**
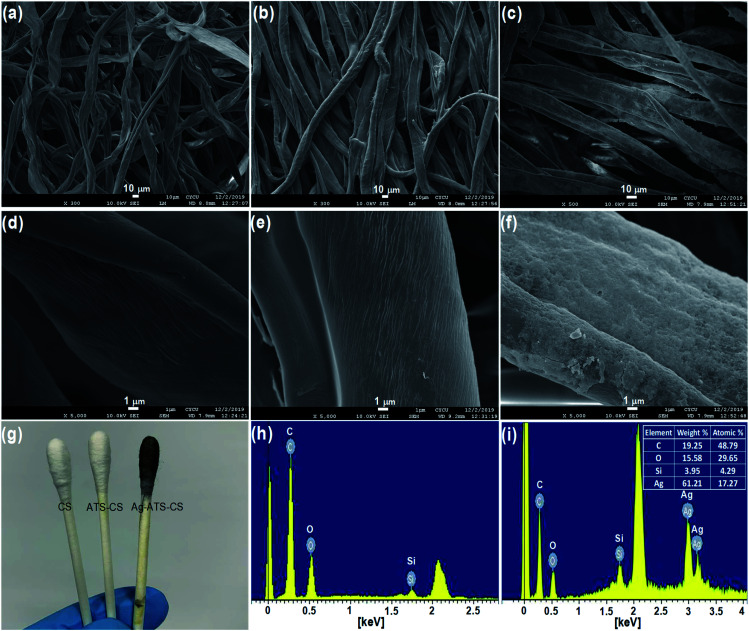
SEM images of (a, d) the pristine CS, (b, e) CS-ATS and (c, f) CS-ATS-Ag. (g) Photograph of the pristine CS, CS-ATS and CS-ATS-Ag. EDS spectrum of (h) CS-ATS and (i) CS-ATS-Ag.

To demonstrate morphologic changes of the pristine cotton swabs after respective surface modification, SEM characterization of the pristine CS, CS-ATS and flexible CS-ATS-Ag cotton swabs was performed, as shown in Fig. 3. As can be seen from Fig. 3(a) and (d), the cotton fibers inside the pristine cotton swabs revealed a relatively smooth surface without an obvious nanostructure, while after modification with ATS, the cotton fibers still maintained the cellulose fabric structure, and the surface of the fibers became much more rough (Fig. 3(b) and (e)). Fig. 3(c) shows a photograph of the flexible CS-ATS-Ag cotton swabs fabricated by assembling AgNPs on cotton fibers. As shown in Fig. 3(d)–(f), SEM images recorded at higher magnifications indicated that the surface of the flexible CS-ATS-Ag cotton fibers seemed to be hierarchical, with a greater roughness than the CS-ATS cotton swabs, due to AgNP implantation on the surface. Then, we characterized the detailed features of the flexible CS-ATS-Ag cotton fibers by SEM, as shown in Fig. 3(f). Clearly, AgNPs were assembled uniformly and densely on the microscale fibers of the cotton fibers without any signs of large-scale aggregation. Fig. 3(g) shows a photograph taken during assembly, wherein the pristine CS and CS-ATS cotton swabs initially appeared colorless on the cotton. Soaking of the activated CS-ATS cotton swabs in AgNP solution and drying of the flexible CS-ATS-Ag cotton swabs made them appear greyish due to the formation of AgNP clusters. Fig. 3(h) shows the EDX profile of the CS-ATS cotton swabs fabricated by assembling ATS on cotton fibers. The profile showed strong signals for C, O and Si, which may have originated from the ATS bound to the surface of the cotton fibers, indicating the presence of ATS. Fig. 3(i) shows an EDX profile of the flexible CS-ATS-Ag cotton swabs fabricated by assembling AgNPs on CS-ATS cotton fibers. The profile showed strong signals for Ag, along with a weak Si peak, which may have originated from the AgNPs that were bound to the surface of the CS-ATS cotton fibers, indicating the presence of AgNPs. According to the qualitative EDX analysis, the presence of C (19.25%), O (15.58%), Si (3.95%), and Ag (61.21%), the major components of ATS and AgNPs, confirmed the presence of ATS and AgNPs in the flexible CS-ATS-Ag cotton swabs. It was verified that the AgNPs were evenly deposited on the whole surface of the CS-ATS cotton fibers (Fig. 3(f)).

### 3.3. Enhancement factor of the flexible CS-ATS-Ag cotton swabs

**Fig. 4:**
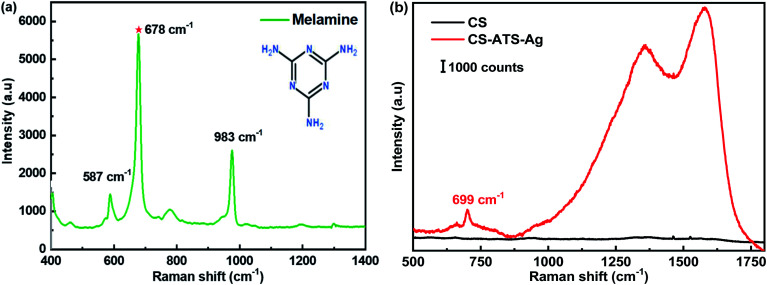
(a) Raman spectra of melamine (b) Raman spectra of modified CS-ATS-Ag cotton swabs before and after soaking in melamine solution.

Fig. 4(a) presents the Raman spectrum of the melamine powder, and sharp and distinct peaks at 587 cm^−1^, 678 cm^−1^ and 983 cm^−1^ were observed. The most prominent peak at 678 cm^−1^ was assigned to the ring-breathing II mode of the C atoms of melamine. The second most prominent peak at 983 cm^−1^ was assigned to the ring-breathing I mode of the triazine ring of melamine.^1^Fig. 4(b) shows the Raman spectra of the cotton swabs before and after modification with AgNPs. We used melamine, which has an obvious Raman characteristic peak at 699 cm^−1^, to evaluate the SERS enhancement effects of the two different types of cotton swab. The pristine CS showed no Raman signal. The SERS signal of melamine obtained from the flexible CS-ATS-Ag cotton swabs after AgNP modification confirmed that the CS-ATS-Ag cotton swab substrate was prepared successfully. The Raman spectrum of the flexible CS-ATS-Ag cotton swabs was obviously changed as compared with the solid Raman spectrum of melamine. The feature peak of melamine solution on the flexible CS-ATS-Ag cotton swabs at 699 cm^−1^ shifted nearly 21 cm^−1^ relative to the peak (678 cm^−1^) in the Raman spectrum of solid melamine. This may be attributed to the effect of the flexible CS-ATS-Ag cotton swab substrate of AgNPs. After mixing with colloidal AgNPs, the feature peak of melamine was red-shifted to 699 cm^−1^ in the Raman spectrum. It was speculated that the enhancement factor was directly related to both surface plasmon resonance (SPR) and chemical molecular enhancement *via* charge transfer between the AgNP surface and the adsorbate.^37^

### 3.4. Sensitivity of the flexible CS-ATS-Ag cotton swabs

**Fig. 5:**
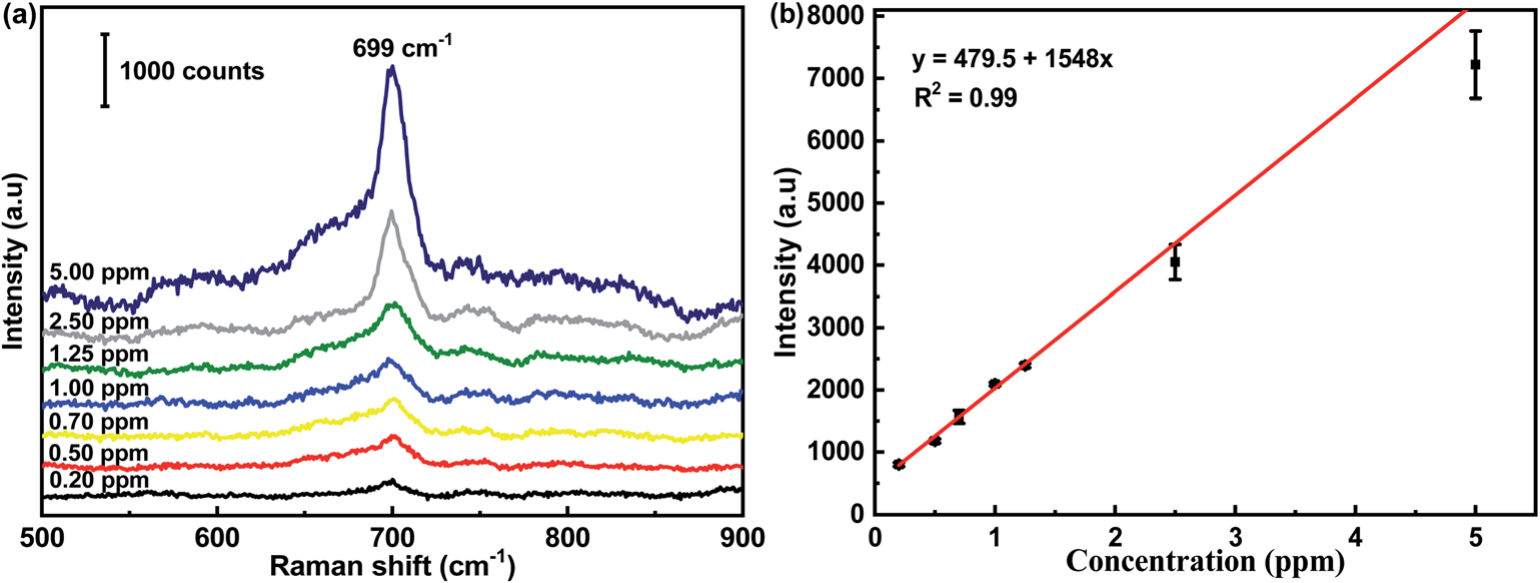
(a) SERS spectra of melamine with varied concentrations (0.2 ppm to 5.0 ppm) acquired from CS-ATS-Ag. (b) The Raman intensity measured at 699 cm^−1^ was plotted as a function of melamine concentrations.

The SERS spectra of melamine at concentrations ranging from 0.20 ppm to 5.00 ppm are presented in Fig. 5(a). The most prominent peak at 699 cm^−1^ in the SERS spectra of melamine was chosen as the feature band, which was attributed to the unique ring-breathing II mode of the C atoms of melamine.^38^ To further verify the linear relationship in quantitative detection, three random points were selected on each substrate to record a SERS spectrum. The intensity of the characteristic peak at 699 cm^−1^ was plotted on the ordinate in order to analyze its linear relationship with the concentration of melamine. It can be seen that the intensity of the representative peak of melamine declined monotonously with the decrease in melamine concentration. In addition, a close linear relationship existed between the intensity of the SERS peak at 699 cm^−1^ and the melamine concentration, as shown in Fig. 5(b). The linear regression equation was *y* = 479.5 + 1548*x* (669 cm^−1^, *R*^2^ = 0.99), which provided a calibration for the quantitative detection of melamine. The limit of detection for melamine using the flexible CS-ATS-Ag cotton swabs was 0.2 ppm, which was below the detection limit that is legislated by the European Union (EU) and the Food & Drug Administration (FDA). In addition, Table 1 compares the analytical performances of various SERS sensors for the detection limit and enhancement effects of melamine, indicating that our SERS sensors were comparable with other SERS sensors reported in literature.

**Table 1:** The analytical performances of various SERS sensors for the detection limit and enhancement effects of melamine were compared

Entry	SERS substrates	Enhancement effects		Targeted analytes	Ref.
		Limit of detection	EF (a.u.)		
1	Ag/PET	10^−4^ M	—	Melamine	39
2	Ag/SiO_2_ filter paper	0.17 mg L^−1^		Melamine	40
3	AgNPs/MoS_2_/pyramidal polymer	10^−6^ M	—	Melamine	41
4	AgNP/filter paper	1 ppm	—	Melamine	42
5	AgNPs/HMM	10^−7^ M	1.72 × 10^8^	Melamine	43
6	NFC/AuNP	1 ppm	—	Melamine	44
7	Ag NC array	0.01 ppm	1.02 × 10^5^	Melamine	45
8	Au@CS	1.5 mg kg^−1^	1.6 × 10^5^	Melamine	46
9	Ag/PPy@PEDOT:PSS	6.4 ng mL	4.4 × 10^2^	Melamine	47
10	AgNPs/filter paper	10^−7^ M	2.2 × 10^8^	Melamine	48
11	Au-ZZF	0.39 μM	1.37 × 10^7^	Melamine	49
12	Ag/cicada	10 mg L^−1^	—	Melamine	50
13	AgNPs/ATS	0.2 ppm	—	Melamine	This work

### 3.5. Reproducibility of the flexible CS-ATS-Ag cotton swabs

**Fig. 6:**
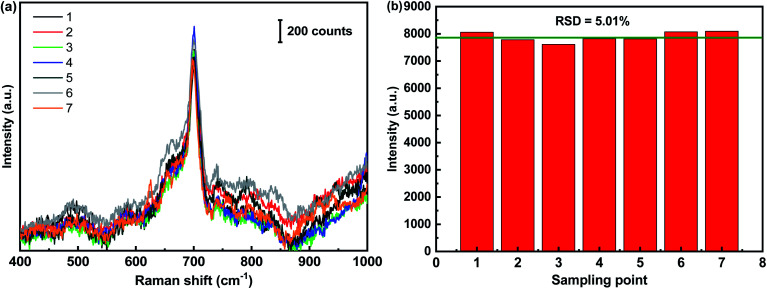
Reproducibility of the CS-ATS-Ag. (a) SERS spectra of melamine (5 ppm) measured on CS-ATS-Ag from 7 random spots. (b) The band at 699 cm^−1^ was used for determination of the signal reproducibility of the substrate.

The reproducibility of SERS signals from the flexible CS-ATS-Ag cotton swabs is an extremely important property for a simply-constructed and multifaceted analytical tool. In order to verify the reproducibility of the flexible CS-ATS-Ag cotton swabs, we randomly selected 7 spots on the surface of the swabs and presented the spot-on-spot intensity variation of the characteristic peak at 699 cm^−1^ (Fig. 6(a)). This revealed that an excellent SERS signal reproducibility was achieved, and the relative standard deviation (RSD) of the melamine peak at 699 cm^−1^ was approximately 5.01% (Fig. 6(b)), indicating the high reproducibility and uniformity of the substrate, which was consistent with the SEM results (Fig. 3(f)). The simplicity of the fabrication strategy and inherent homogeneity of the cotton fibers were the main reasons for the high level of uniformity.

### 3.6. Stability of the flexible CS-ATS-Ag cotton swabs

**Fig. 7:**
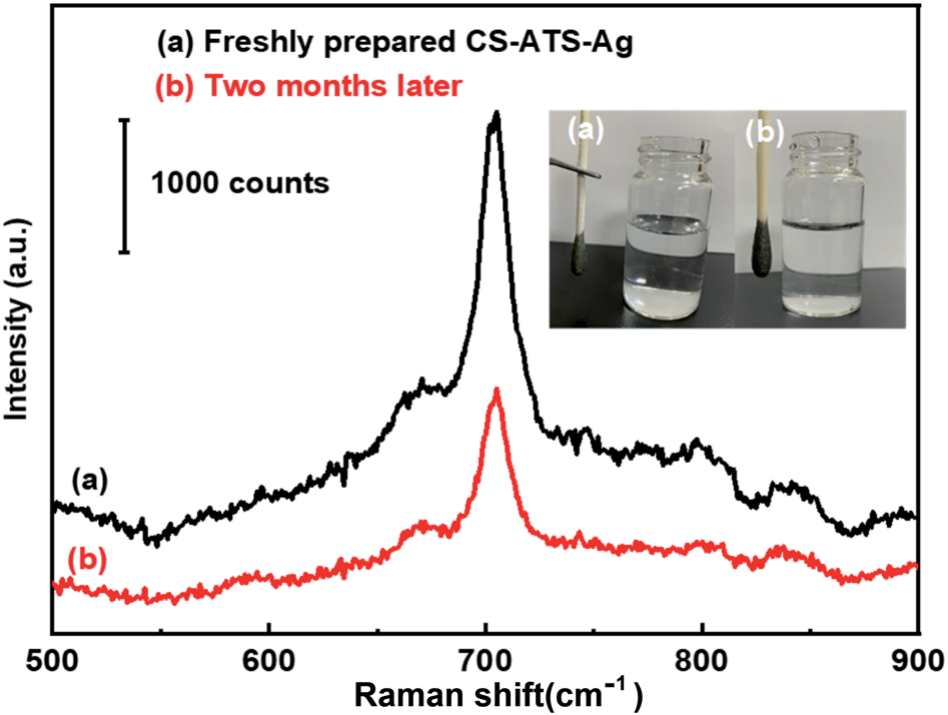
Stability of the CS-ATS-Ag. (a) Freshly prepared and (b) two months later.

As shown in the illustration in Fig. 7, we observed that once the AgNPs were assembled on the CS-ATS cotton swabs, vigorous rinsing with ultrasonication in water did not remarkably change the surface morphology of the swabs. This highlights the excellent stability of the flexible CS-ATS-Ag cotton swabs in liquid environments. The uniform and irreversible assembly of the flexible CS-ATS-Ag cotton swabs was primarily attributed to the electrostatic interaction between Ag^+^ ions and the amino group modified on CS-ATS cotton fibers, leading to AgNP adsorption and diffusion on the flexible CS-ATS-Ag cotton swab surface.^51,52^ Furthermore, the SERS stability of the CS-ATS-Ag cotton swabs towards melamine was also investigated in detail. The Raman spectrum of newly prepared and the CS-ATS-Ag cotton swabs aged at room temperature for two months were compared. As shown in Fig. 7, the peak intensity decreased nearly half with the same characteristic peaks position. Although the peak intensity decreased nearly half, it still runs well enhancement effect.

### 3.7. Chemical analysis App development

**Fig. 8:**
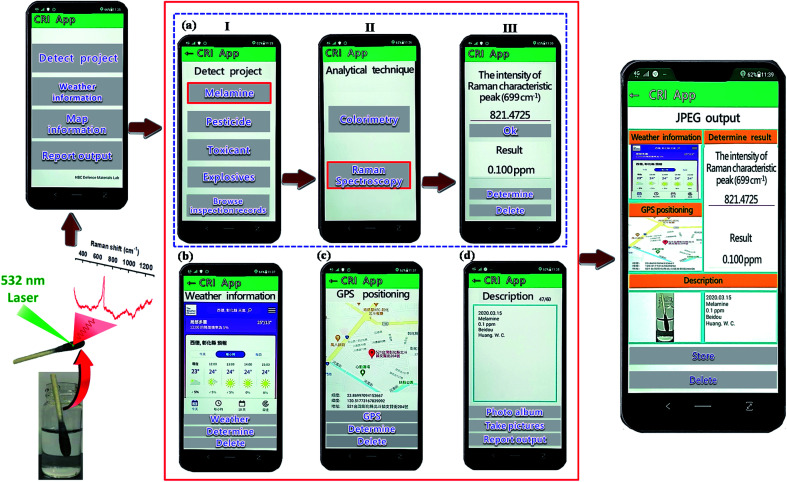
Cell phones can be used for data collection and to push data to a website where data is displayed on a map.

We successfully developed Chemical analysis App application software, using Android studio programming language. Our App has four primary functions: (i) conversion of data, (ii) GPS positioning information query, (iii) weather information query, and (iv) test result reporting and transmission. A schematic of our proposed process is presented in Fig. 8. First, the user can click “Detect Project” to select the detection type (Fig. 8(a)I), and then click “Melamine” to select the appropriate analytical technique (Raman spectroscopy) and obtain the working curve (Fig. 8(a)II). When the user wishes to measure an unknown concentration of melamine, they enter a peak value of the Raman spectrum, click “OK”, and the concentration of melamine is displayed on the screen (Fig. 8(a)III), providing the user with immediate data. The testing results, along with other information (*e.g.*, position, weather, image and description), can be wirelessly transmitted to a central website and made available to the relevant authorities and the general public. The goal of realizing instant data transmission and timely processing was therefore achieved.

## 4. Conclusions

The results showed that the flexible CS-ATS-Ag cotton swabs exhibited an excellent SERS performance, with a detection limit for melamine of 0.2 ppm, and had a high reproducibility, the intensity variation of the SERS peaks being within 5.01%. The flexible CS-ATS-Ag cotton swabs achieved rapid and timely SERS detection of melamine, not only by swabbing of a solid surface, but also by adsorption in solution. More importantly, the flexible CS-ATS-Ag cotton swabs are disposable and portable. Further, our Chemical analysis App transmits test results to a central website, and communicates detection information (*i.e.*, position, weather, image, description and detection results) to food safety officers, providing critical data for policy-makers and the general public. The goal of realizing instant transmission, timely processing, high sensitivity, portability and low cost was therefore achieved.

## Conflicts of interest

The authors declare that there is no conflict of interest in this paper.

## Supplementary Material
